# A Pilot Cross-Sectional Study on Oral Health and Nutritional Status of Institutionalized Older Adults: A Focus on Sarcopenia

**DOI:** 10.3390/ijerph182413232

**Published:** 2021-12-15

**Authors:** Luca Aquilanti, Sonila Alia, Sofia Pugnaloni, Lorenzo Scalise, Arianna Vignini, Giorgio Rappelli

**Affiliations:** 1 Department of Clinical Specialistic and Dental Sciences, Università Politecnica delle Marche, Via Tronto 10/A, 60126 Ancona, Italy; l.aquilanti@pm.univpm.it (L.A.); s.alia@pm.univpm.it (S.A.); s.pugnaloni@pm.univpm.it (S.P.); g.rappelli@staff.univpm.it (G.R.); 2 Department of Industrial Engineering and Mathematical Sciences, Università Politecnica delle Marche, Via Brecce Bianche 12, 60131 Ancona, Italy; l.scalise@staff.univpm.it; 3 Dentistry Clinic, National Institute of Health and Science of Aging, IRCCS INRCA, Via Tronto 10/A, 60126 Ancona, Italy

**Keywords:** nutritional status, oral health, mastication, sarcopenia, ageing

## Abstract

The global population aged over 60 will double by 2050. This pilot cross-sectional study aims at evaluating nutritional and oral health status and the prevalence of sarcopenia in older adults living in an Italian residential aged care facility. Thirty-two adults aged ≥65 years were included. Individual sociodemographic data and nutritional and oral health data were collected. For sarcopenia diagnosis, muscle mass, physical performance, muscle strength and anthropometric parameters were recorded. Participants underwent a nutritional screening and a dental examination. Mini Nutritional Assessment and masticatory mixing ability test were performed. The results showed that men recorded a hand strength significantly higher than that of women, 25.5 ± 7.2 Kg vs. 12.8 ± 5.9 Kg (*p* < 0.01), respectively. Gait speed test showed that only 20.8% of the participants had a speed of more than 0.8 m/s. A strong negative correlation between masticatory performance and the number of missing teeth was detected (r = −0.84, 95% C.I. [−0.92; −0.69], *p* < 0.01). Overall, a high percentage of institutionalized older adults were diagnosed as being sarcopenic. Poor oral health in older adults is a major general health problem as it may restrict both food selection and nutrient intake, representing a risk factor for sarcopenia, although longitudinal studies are needed to confirm this relationship.

## 1. Introduction

The global population aged over 60 is expected to double in the coming years, implying the need to promote healthy longevity and ageing while avoiding diseases and functional disability [1,2]. On one hand, the increase in life expectancy involves potential opportunities for society and families; on the other, all those opportunities are directly associated with health. The World Health Organization (WHO) has defined the term “healthy ageing” as the capacity of maintaining a functional status that enables well-being in older age [3]. Thus, constructive efforts are needed in order to foster healthy ageing, especially in the institutionalized elderly.

The clinical condition of sarcopenia is one of the most challenging aspects of an ageing population. Sarcopenia is described as a progressive and generalized skeletal muscle disorder that is related to an increased probability of unfavourable consequences such as falls, fractures, physical disability and mortality [4]. According to the European Working Group on Sarcopenia in Older People 2 (EWGSOP2), muscle strength, muscle quantity or quality and physical performance are the three diagnostic criteria on which a diagnosis of sarcopenia is made [4]. This condition is deemed to be a major public health issue in the elderly, and one which also affects their quality of life [5]. Many risks factors can enhance the onset of sarcopenia, such as ageing, sedentary lifestyle, hospitalization, immobilization, chronic diseases, inflammation, metabolic derangements, oxidative stress and nutrition. The latter influences muscle activity by affecting myocyte homeostasis and energy metabolism, and takes part in the pathogenesis of sarcopenia [6]. In addition to the physiological reduced intake of energy and nutrients that occurs with ageing, other factors play an important role in determining this phenomenon (e.g., loneliness and surroundings, functional ability and autonomy loss, and financial status) [7]. In particular, malnourishment and a decline in physical function are especially noted in institutionalized older adults [8].

Additionally, tooth loss could worsen nutritional status, as it is associated with the risk of malnutrition or with malnourishment itself [9]. It was stated that poor oral health status is a strong predictor of the inception of adverse health outcomes, including mortality among the community-dwelling elderly [10]. An increased risk of losing food micronutrients is associated with the inability to shred and chew food properly, excluding and/or overcooking some basic foods [11]. A reduced masticatory function could be responsible for inadequate nutrition but this food restriction could also be due to many other causes, including reduced gustatory and olfactory perception, as well as economic and psychological factors [11,12,13,14]. However, it is not clear if changes in dietary behaviours and the consumption of certain foods could influence the onset of conditions leading to edentulism and to a further reduction of the masticatory function. When dealing with aging, the interception of oral health diseases, resulting in a decrease in oral function, should be pursued in order to promote a healthy life and prevent important risk factors for malnourishment and sarcopenia [15].

Considering the high risk of diagnosing sarcopenia among community-dwelling and institutionalized elderly, the evaluation of factors associated with such conditions should be pursued in order to lessen their adverse sequelae on health. In fact, the age-related sarcopenia, which affects the masticatory muscles, may be worsened by tooth loss and poor oral conditions [16]. This said, the aim of this pilot cross-sectional study is to evaluate oral health conditions and the prevalence of sarcopenia in subjects living in an Italian residential aged care facility.

## 2. Materials and Methods

The present pilot study enrolled 65 years and over adults living in “Casa di Riposo Grimani Buttari”, Osimo (Ancona), Italy, who underwent a comprehensive geriatric health examination from December 2018 to May 2019. The study was conducted according to the guidelines of the Declaration of Helsinki and approved by the Institutional Review Board of Dentistry Clinic, Università Politecnica delle Marche, Ancona, Italy (ODO-EXP-107/18, 19 June 2018). Written, informed consent was obtained from all participants.

Individual sociodemographic data and general health data were recorded for all participants. Included subjects had to be ≥65 years old and compliant. Subjects were excluded if they suffered from neurodegenerative conditions and were not compliant. The Institute’s healthcare team selected participants on the basis of subjects’ medical history.

For the definition of sarcopenia, the recommendations of the EWGSOP2 were followed [4]. For the diagnosis of sarcopenia, several measurements were recorded, such as assessment of muscle mass, physical performance, or muscle strength.

### 2.1. Muscle Mass, Performance and Strength Analyses

Muscle mass was measured by using a bioimpedance analysis performed by DF50 Body Composition Analyzer (ImpediMed, Brisbane, Australia; accuracy: ±0.5%).

Briefly, bioimpedance analysis measures the body’s resistance to the flow of a low-intensity (800 μA) and high-frequency (50 kHz) electric current. Bioimpedance was carried out by placing a pair of electrodes, which are connected to the measuring instrument, on the back of the hand and another pair on the back of the foot. Impedance (Z), Resistance (R), Phase Angle (PhA) and Reactance (Xc) were evaluated. Patients were placed in a supine position on a medical examination couch so that the body was parallel to the ground. The patients’ arms had to be distanced from the torso at an angle of about 30° and their legs had to be distant from each other at an angle of about 45°. Four electrodes were then positioned; two electrodes on the back of the hands and feet and another two on the bony prominence of the wrists, and between the medial malleolus and the lateral ankle [17].

To evaluate muscle performance, reference was made to the usual gait speed test, as it has proven to be rapid, safe, and very reliable. To perform the test, patients were asked to follow a straight course of 4 m at their usual speed and the journey time was measured in seconds (a single cut-off speed ≤0.8 m/s).

To evaluate physical strength, the hand grip strength test was performed. This can be used as a key indicator both in the evaluation of sarcopenia and in regard to the phenotypes of fragility [18]. A digital grip strength dynamometer (Camry Scale, Zhongshan, China; resolution: 0.98 N) was used for the hand grip test to evaluate muscle strength. In order to determine the dominant hand, subjects were asked what hand they actively used. Subjects sat on a chair, with their elbows on the table and arms parallel in a 90-degree flexion; measurements were made three times with 1 min rest periods. The maximum value of the three consecutive measurements was recorded. Measurements below 27 Kg (265 N) for men and below 16 Kg (157 N) for women were counted as low muscle strength [19].

### 2.2. Anthropometric Measurements

With regard to anthropometric measurements, body mass was measured with resolution of ±0.1 Kg on a balance beam scale with the subject dressed in indoor clothing without shoes. The nursing home’s latest recorded weight was used for participants who could not stand, and it was usually up to one month old. Height was measured to the nearest 0.1 cm using a wall-mounted stadiometer (Seca, Hamburg, Germany), or the alternative measurements of knee height and ulna length were used. These values were utilized to calculate the body mass index (BMI). BMI was calculated as weight (in kilograms) divided by square of height (in meters). Subjects were divided into different groups according to BMI and all-cause mortality risk [20]. In particular, three different groups were identified: augmented risk of mortality if BMI was <23, reference range if BMI was 23–30, and increased risk of mortality if BMI was >30.

Trained staff used a metric band (resolution: ±1 mm) to record the circumference of the biceps (BC), measured at midpoint between the olecranon process and the acromion with the participant’s arm bent 90° at the elbow, and waist circumference (WC), measured according to a horizontal plane parallel to the floor, at the natural waist or narrowest part of the torso with a precision of ±0.5 cm.

### 2.3. Nutritional Assessment

Subjects were asked to participate in both a qualitative and quantitative food interview for the assessment of nutritional status. The nutritional status was assessed using the Mini Nutritional Assessment (MNA). MNA can be completed by nurses, and it is used to evaluate the nutritional status of institutionalized older people [21].

Overall, MNA includes 18 items with an ordinal scale for response and their sum ranges from 0 to 30. Subjects are classified in MNA categories on the basis of the total score: an adequate nutritional status is considered as >23.5 points, a risk of malnourishment as 17–23.5 points and malnourishment below 17 points [22].

The meals consisted of a continental-style breakfast, lunch which included a choice between two hot dishes, and fruit, and dinner consisting of soup and either a hot or cold food option and dessert, and three daily snacks. The diet was developed by a well-trained dietitian (S.A.) whose task was also to communicate with the kitchen staff.

### 2.4. Dental Examination

**Figure 1:**
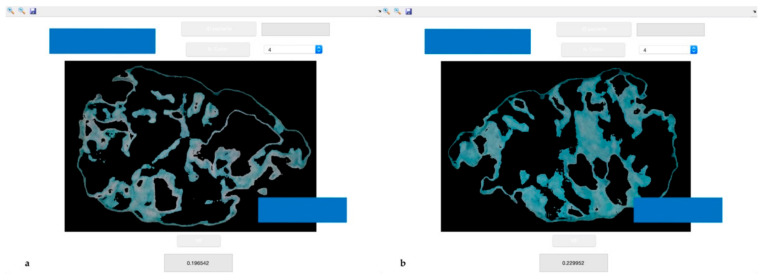
Example of the digital analysis of a chewing-gum bolus, side A (**a**) and side B (**b**). The software used automatically analyses the images and gives as output the ratio of the mixed portion of the sample.

All patients underwent dental examination. The number of missing teeth and of occluding pairs were recorded, as well as Decayed Missing Filled Teeth (DMFT), Full Mouth Plaque Score (FMPS), Periodontal Screening and Recording (PSR) and self-reported masticatory difficulties using a 0 to 10 visual analogue scale (VAS). Masticatory performance was assessed using a mixing ability test. In short, the test involves the use of two-coloured chewing gums (Hue-check Gum^®^, Orophys GmbH, Muri b. Bern, Switzerland). Each sample was chewed for 20 chewing cycles, as this number of strokes allows us to assess the masticatory performance. Boluses were collected and inserted between two sheets of transparent plastic, yielding samples of 1 ± 0.1 mm of thickness. Standardized photos were taken from both sides of each bolus, and all the obtained images were processed by computer, analysing the measure of the area of pixels of different colours using the k-means clustering method [23]. At the end of the analysis, the software revealed the ratio between mixed and unmixed areas of the boluses, discriminating between the different masticatory performances of the subjects. Figure 1 shows an example of the outcome of the sample analysis.

The rationale for using the above-mentioned oral health parameters was their reliability, quickness, and reproducibility. In particular, the assessment of caries index (DMFT) and periodontal health status (PSR) was performed as caries and periodontal diseases are the main causes of tooth loss in adulthood. FMPS index was carried out in order to evaluate how good the oral hygiene procedure performed by the enrolled subjects were. In addition, masticatory function was evaluated both subjectively (self-reported masticatory difficulties) and objectively (two-colour mixing ability test).

### 2.5. Statistical Analysis

Data were analysed using R statistical software (R Foundation for Statistical Computing, Vienna, Austria). The normal distribution of continuous variables was tested by the Kolmogorov–Smirnov test. For continuous data, a Mann–Whitney test and a t-student with Welch correction test were used. Chi-square test was used for significance of associations with categorical variables. Pearson correlation coefficient was used to assess correlations between the tested variables. Data were expressed as Mean ± SD. A value of *p* < 0.05 was considered statistically significant.

## 3. Results

During the period from December 2018 to May 2019, 190 subjects attended “Casa di Riposo Grimani Buttari”, Osimo (Ancona), Italy. Out of 190 residents, 16.8% (n = 32) of the subjects met the inclusion criteria and were enrolled in this study. These included eight men and 24 women, aged over 65 years (mean ± SD age: 86.7 ± 5.7). The patients’ medical histories showed the presence of some systemic diseases, such as hypertension (15 subjects), osteoarthrosis (10 subjects), heart failure (3 subjects), chronic obstructive pulmonary disease (5 subjects), and diabetes (7 subjects). Table 1 summarizes both the sociodemographic and clinical data, reporting statistically significant differences between men and women.

Through the anamnestic interview, 59.4% (n = 19) of the participants declared that they were non-smokers, 31.3% (n = 10) were former smokers, 6.3% (n = 2) used to smoke less than 10 cigarettes or equivalent per day, and 3.0% (n = 1) used to smoke more than 10 cigarettes or equivalent per day. A total of 78.1% declared having no drinking habits and 21.9% (n = 7) declared that they used to consume less than or equal to 2 daily units of alcoholic drink or equivalent. No one declared drinking more than 2 daily units of alcoholic drink equivalent. A total of 28.1% (n = 9) declared brushing their teeth more than once a day, while 71.9% (n = 23) did not perform oral hygiene procedures or only once a day (*p* < 0.01). All the participants were undergoing polypharmacy with a mean ± SD number of drugs per day per subject of 8.2 ± 3.2.

### 3.1. Muscle Mass, Performance and Strength Analyses

The results showed that the men recorded a mean strength of 25.5 ± 7.2 Kg (250 ± 70.6 N), significantly higher than that of women, 12.8 ± 5.9 Kg (126 ± 57.8 N), (*p* < 0.01). In the present study, 62.5% of the male population scored less than 27 Kg and 87.5% of women scored less than 16 Kg. With regard to gait speed, 8 of the 32 participants did not perform the test because they were unable to walk. Overall, a mean speed of 0.58 ± 0.25 m/s was recorded. Pearson’s correlation coefficient indicated weak positive correlation between gait speed test results and strength (r = 0.48, 95% C.I. [0.10; 0.74], *p* < 0.05). A weak negative correlation was shown between WC and gait speed test results (r = −0.47, *p* < 0.05, 95% C.I. [−0.73; −0.08]). According to the bioimpedance analysis, no statistically significant differences were assessed between men and women Z, R, Xc and PhA values (*p* > 0.05). The mean values (mean ± SD) of bioimpedance parameters were 500.3 ± 98.2, 499.0 ± 99.3, 33.8 ± 7.9 and 4.1 ± 2.0, respectively. In particular, PhA was very low in 19 subjects (PhA = 2–4°), while in 11 patients a PhA range value between 4–6° was recorded. With regard to MNA scores and bioimpedance, a statistically significant difference was assessed between both R and Z scores in participants at risk of malnourishment and those with an adequate nutritional status (*p* < 0.05). When assessing Pearson’s correlation coefficient among bioimpedance parameters and the other anthropometric data, moderate negative correlations were detected between WC and R and between WC and Z (r = −0.59, 95% C.I. [−0.78; −0.31], *p* < 0.01).

EWGSOP2 operational definition of sarcopenia [4] defines low muscle strength as the primary criterion of sarcopenia, followed by low muscle quantity and quality and low physical performance. In particular, sarcopenia is probable when low muscle strength is detected. A sarcopenia diagnosis is confirmed by the presence of low muscle quantity or quality. When low muscle strength, low muscle quantity or quality and low physical performance are all detected, sarcopenia is considered severe. Taking this into account, the first criterion was detected in 37.5% of men (n = 3) and in 29.2% of women (n = 7) (probable sarcopenia diagnosis). Low muscle strength associated with low muscle quantity/quality was observed in 25% of men (n = 2) and in 12.5% of women (n = 3) (sarcopenia diagnosis). Finally, the whole criteria were assessed in none of the men and in 45.8% of women (n = 11) (severe sarcopenia diagnosis).

### 3.2. Anthropometric Measurement

**Figure 2:**
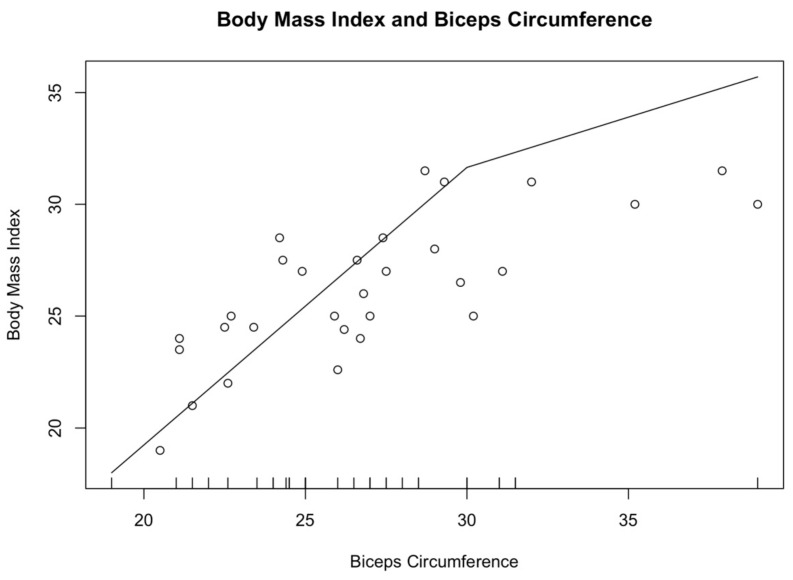
Association between Body Mass Index and Biceps Circumference. The figure graphically outlines the association between BMI and BC: with the increase in the circumference of biceps, also the Body Mass Index increases (R^2^ = 0.63).

The participants were divided into groups according to their BMI. Eight subjects who recorded a BMI score < 23, 17 were within the normal weight range (23 ≤ BMI ≤ 30), and seven recorded a BMI higher than 30. Men had a higher WC than women, 101.8 ± 5.5 cm vs. 95.5 ± 11.6 cm, respectively. Conversely, women recorded a higher BC than the men one (26.6 ± 3.9 cm vs. 26.3 ± 4.1, respectively). Significant differences were not detected between male and female parameters (*p* > 0.05). According to Pearson’s correlation coefficient, a moderate positive correlation was found between BMI and BC (r = 0.77, 95% C.I. [0.58; 0.88], *p* < 0.01). Figure 2 graphically outlines the association between BMI and BC, with the increase in the circumference of biceps and in the Body Mass Index (R^2^ = 0.63). Similarly, Pearson’s correlation coefficient showed a moderate positive correlation between BMI and WC (r = 0.71, 95% C.I. [0.43; 0.87], *p* < 0.01).

### 3.3. Nutritional Assessment

The results of MNA revealed that 40.6% (n = 13) had an adequate nutritional status (score ≥ 24), 59.4% (n = 19) were at risk of malnourishment (score between 17 and 23.5), while no subjects were malnourished (score < 17). In particular, 15 women and five men recorded a MNA score between 17 and 23.5, and nine women and five men had a score ≥ 24.

### 3.4. Dental Examination

**Figure 3:**
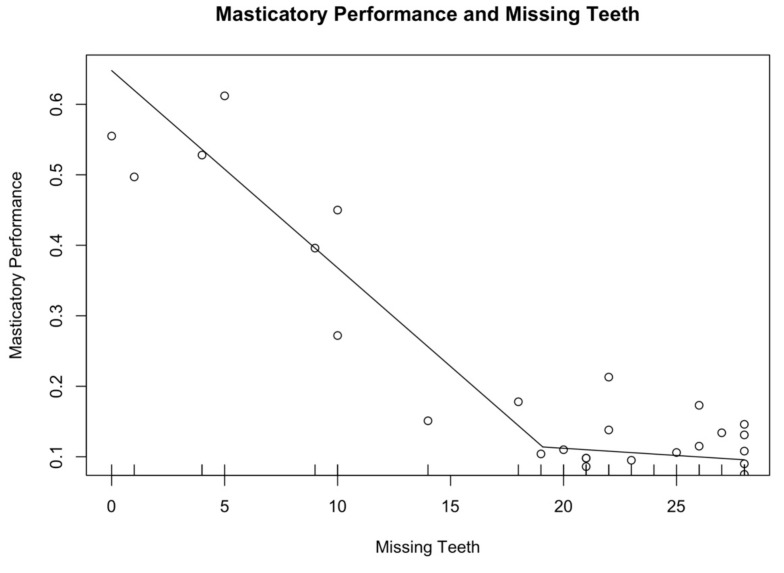
Association between Masticatory Performance and Missing Teeth. The figure graphically represents the association between masticatory performance and the number of missing teeth: the line drops dramatically to 19 missing teeth, then the line gently declines (R^2^ = 0.87).

Dental examination revealed that 15.6% (n = 5) of the participants had more than 20 teeth (mean ± SD: 25.2 ± 2.2), while 84.4% (n = 27) had a mean ± SD of 5.7 ± 5.9 missing teeth. Overall, a mean ± SD of 8.8 ± 9.0 teeth and 3.6 ± 4.4 occluding pairs were detected. A mean masticatory performance of 0.28 ± 0.20 was calculated among the participants. DMFT scores showed a mean score of 20.1 ± 8.3. When considering PSR index, a mean value of 3.1 ± 1.0 was assessed in the whole study sample. Significant differences were not shown between men and women (*p* > 0.05). FMPS was assessed for each participant, considering six surfaces per tooth and having set 20% as cut-off. Results showed that four subjects had an FMPS lower than 20% and 28 had a FMPS equal or higher than 20% (*p* = 0.001). Finally, seven subjects had neither fixed nor removable dental prostheses, while eight had fixed dental prostheses (bridges and crowns both on natural teeth and dental implants), 22 removable dental prostheses (both partial and total) and five had both. Seventeen (53.1%) participants reported difficulties in chewing and 65.6% (n = 21) prosthodontic discomfort. According to Pearson’s correlation coefficient, a strong negative correlation was detected between masticatory performance and number of missing teeth (r = −0.84, 95% C.I. [−0.92; −0.69], *p* < 0.01). Figure 3 graphically represents the association between masticatory performance and the number of missing teeth: the line drops dramatically to 19 missing teeth, then the line gently declines (R^2^ = 0.87).

When assessing the correlation between the number of occlusal units and masticatory performance a fairly strong positive correlation was recorded (r = 0.85, *p* < 0.01, 95% C.I. [0.72; 0.93]). A moderate positive correlation was shown between VAS and MP (r = 0.70, 95% C.I. [0.47; 0.85], *p* < 0.01). When assessing possible associations among MP and the other studied parameters no significant associations were found (*p* > 0.05). Overall, poor oral health was assessed in the enrolled subjects.

## 4. Discussion

The present pilot cross-sectional study was performed on a sample of 32 subjects, who were resident in an Italian residential aged care facility. Overall, 81.3% (n = 26) of the sample were diagnosed with probable, confirmed, or severe sarcopenia. Moreover, poor oral health status was assessed among participants in terms of poor oral hygiene, low masticatory performance, and low chewing and prosthetic comfort. MNA showed that almost 60% of the residents were at risk of malnourishment.

The global population is ageing. In Australia and the USA, the number of elderly people living in residential aged care facilities has greatly increased [24,25]. Moving into residential aged care facilities is more common among women than men. This datum is in accordance with a recent systematic review [26], and with the results of the present study, where the number of men and women differed statistically (*p* < 0.05). An explanation of this could lie in the fact that women have a higher life expectancy than men, exposing women to a higher risk of incurring debilitating diseases and of requiring daily health care.

As stated elsewhere, a significant association was found between nutritional risk and quality of life in the elderly [27]. In particular, malnourishment, low physical health, and sensory deficiencies are the factors that are most strongly associated with a worsening of health in elderly [28]. According to the results of the present study, the 59.4% of the sample recorded a MNA score that indicates the risk of malnourishment. Women were more commonly affected than men, with a female/male ratio of 1.3, in agreement with a previous study [29]. Nutritional status has a role in the pathogenesis of sarcopenia. EWGSOP2 guidelines defines three criteria on the basis of which sarcopenia is operatively diagnosed. Probable sarcopenia is identified when low muscle strength is assessed. Diagnosis is confirmed if low muscle quantity or quality is evaluated, and sarcopenia is considered severe if low physical performance is added to criterion one and two. A great variety of techniques are available to assess muscle quantity or mass [30]. Although Computed Tomography and Magnetic Resonance Imaging are considered to be the gold standards for the assessment of muscle quantity or mass, these methods are not commonly used in primary care due to lack of portability, the requirement for highly trained personnel and high equipment costs [31]. Dual-energy X-ray absorptiometry is more commonly used. However, its disadvantage is that the instrument is not portable, and measurements can also be influenced by the hydration status of the patient [32]. Bioimpedance analysis was used to assess muscle mass. It is a non-invasive method that analyses tissue properties and gives reliable information about body composition by transmitting a series of alternating electric currents through the body. This method is not expensive, requires no specialized staff and is relatively easy to use in clinical practice, either on outpatient subjects or on hospitalized patients [31]. Moreover, reference values have been established for the elderly. Low PhA values suggest cell death or reduced cell integrity, while high PhA values indicate intact cell membranes: PhA was proposed as a parameter for predicting not only clinical outcomes, but also mortality from various diseases, including sarcopenia [17]. Gait speed test was employed to evaluate physical performance. The latter was defined as the objective measurement of the whole-body function related to locomotion. This test is widely used in practice because it is considered a quick, safe and highly reliable test for sarcopenia.

Overall, sarcopenia was not diagnosed in 18.7% of the sample, while probable sarcopenia, confirmed sarcopenia and severe sarcopenia were diagnosed in 31.3%, 15.6% and 34.4%, respectively. These data are in accordance with those available in the literature. The prevalence of sarcopenia is very high among hospitalized older adults and it was found to be directly related to nutritional status and hospital stay time [33]. In the present study, 65.6% of women were diagnosed with probable, confirmed, or severe sarcopenia, compared to 15.6% of men. This result is in accordance with those of Yalcin et al. and Shen et al. but in contrast with those of Landi et al. and Kim and Won [33,34,35,36]. An explanation for this could lie in the fact that several discrepancies in sex distribution could be observed among the studies [26]. Several studies considered sarcopenia as a significant predictor of all causes of mortality among community-dwelling residents. In particular, subjects with a diagnosis of severe sarcopenia and those with deteriorated physical performance had a higher risk of death, thus highlighting the need to immediately intervene [37].

Poor oral conditions are an important indicator of physical frailty, sarcopenia, need for long-term care, and mortality. Poor oral health conditions, swallowing and masticatory problems contribute in part to dietary restrictions and to a poor nutritional status in elderly, increasing the risk of frailty and sarcopenia. Similarly, oral conditions may be influenced both by frailty and sarcopenia, probably through the common burden of inflammation and oxidative stress [38]. Additionally, in the present study, poor oral conditions were assessed among subjects. Masticatory performance was associated both with the number of teeth and the number of occluding pairs (r = 0.84 and 0.85, *p* < 0.01, respectively). Moreover, masticatory performance was significantly lower in subjects with less than 20 teeth than in those with more than 20 (*p* < 0.01), as shown elsewhere [39]. The high prevalence of missing teeth among the participants of the present study is an indicator of unmet dental treatments, in terms of both caries and periodontal disease. As stated by Kassebaum et al., population growth and the increase in life expectancy have determined a dramatic rise in the burden of untreated oral conditions throughout the world. In particular, the loss of natural teeth, as a consequence of periodontal diseases and untreated caries, is the major cause of Disability-Adjusted Life Years (DALYs) due to oral conditions [40]. It becomes crucial to the effective preventive and therapeutic programs pursued in order to retain the natural dentition and masticatory function in old age, contributing to the delay of physical and cognitive decline as well as of dependence loss. The relationship between oral health and low physical activity has been considered in several studies, especially in institutionalized older adults [41]. Additionally, poor oral health has been associated with a faster decline in handgrip strength, which is an important risk factor for sarcopenia [38]. The results of the present study showed that, overall, men had a significantly greater hand-grip strength than women (*p* < 0.01). Nevertheless, 62.5% of the male population and 87.5% of women scored less than the threshold values, which are 27 kg (264.8 N) and 16 kg (156.9 N), respectively. In the sample, no significant associations were detected among masticatory performance and the studied nutritional parameters (*p* > 0.05). In addition, no statistically significant differences were detected between participants at risk of malnourishment and those with an adequate nutritional status, according to the number of missing teeth. A possible explanation of such a result may be the possible adaptation to the physiological and pathological changes that could occur in ageing and specific diet developed by a well-trained dietitian.

The small number of participants enrolled and the exclusion of those subjects with neurodegenerative diseases could be a limitation of this pilot study. Neurodegenerative conditions and the non-compliance of most of the residents of the residential age care facility restricted the sample of this study. In addition, the number of men and women differed statistically (*p* < 0.05); even if the unequal number of men and women may represent a limit of this study, it could be explained by the fact that women have a higher life expectancy than men, exposing women to a higher risk of incurring diseases and disability, thus requiring daily health care and possible hospitalization. Moreover, some methodological issues may have influenced the results of the present report. The cross-sectional design of the study did not allow us to clarify any cause–effect relationships. Furthermore, results may be confounded by unmeasured factors. Being a pilot study, its limited sample size does not allow us to draw definitive conclusions. However, we were able to assess the feasibility and the operational acceptability of the study protocol. Further research is needed and the enlargement of the sample size, as well as the enrolment of other residential aged care facilities, should be pursued in order to more deeply investigate the relationship among oral health, nutritional status and sarcopenia in older adults. In fact, even if oral health is considered a crucial element of general health and well-being, it is often neglected, especially in frail older people, determining the occurrence of adverse health outcomes.

## 5. Conclusions

Within the limitations of this report, a high prevalence of institutionalized older adults diagnosed at risk of developing sarcopenia or of being sarcopenic or severely sarcopenic was shown. Moreover, poor oral conditions were assessed among this kind of subject. Despite the cross-sectional design of this pilot study, and although a clear association was not shown, the impairment of oral function and the diagnosis of sarcopenia may be considered as factors responsible for the worsening of the general health status. A multidisciplinary approach could help to ensure the maintenance of good oral health status and adequate nutrition, preventing and intervening in the multiple factors of sarcopenia that could lead to the worsening of the clinical status, especially in the elderly. Regular diet, specific physical activity and oral health preventive programs should be crucial goals to be pursued. Both the scientific community and policy makers should pay greater attention to the provision of care to older adults.

## Figures and Tables

**Table 1:** Sociodemographic and clinical characteristics of the study population. Data values are expressed as the number of participants for sex and mean and standard deviation for all other variables.

Sociodemographic and Clinical Data	Total	Males	Females	*p*-Value
Sex (n)	32	8	24	0.01
Age (years)	86.7 ± 5.7	83.3 ± 6.0	87.8 ± 5.5	0.07
Drugs (n)	8.2 ± 3.2	9.5 ± 3.2	7.8 ± 3.2	0.32
BMI ^1^ (kg/m^2^)	27.0 ± 5.2	25.8 ± 5.5	27.4 ± 4.9	0.44
Waist Circumference (WC) (cm)	97.1 ± 11.6	101.8 ± 5.5	95.5 ± 11.6	0.19
Biceps Circumference (BC) (cm)	26.5 ± 3.9	26.3 ± 4.1	26.6 ± 3.9	0.80
Hand Grip Test (kg)	16.0 ± 6.8	25.5 ± 7.2	12.8 ± 5.9	<0.01
Four meters Test (sec)	8.0 ± 3.0	7.7 ± 3.2	8.1 ± 2.9	0.85
Gait speed Test (m/s)	0.6 ± 0.3	0.7 ± 0.3	0.6 ± 0.2	0.55
Number missing teeth	19.3 ± 9.0	18.9 ± 9.5	19.4 ± 9.2	0.91
Occluding pairs	3.6 ± 4.4	4.1 ± 4.6	3.4 ± 4.5	0.73
Masticatory Performance (%)	28.1 ± 20.1	32.2 ± 21.4	26.7 ± 20.7	0.54
DMFT ^2^	20.1 ± 8.3	19.4 ± 8.8	20.3 ± 8.4	0.81
PSR ^3^	3.1 ± 1.0	3.4 ± 0.8	2.9 ± 1.0	0.18

^1^ Body Mass Index (BMI); ^2^ Decayed Missing Filled Teeth (DMFT); ^3^ Periodontal Screening and Recording (PSR).

## Data Availability

The data sets generated and/or analysed during the present study are available from the corresponding author on reasonable request.
